# Obstructive Sleep Apnea in Hypertension

**DOI:** 10.7759/cureus.38229

**Published:** 2023-04-27

**Authors:** Shyam C Chaudhary, Pankaj Gupta, K K Sawlani, Kamlesh K Gupta, Abhishek Singh, Kauser Usman, Vivek Kumar, D Himanshu, Ajay Verma, Abhishek B Singh

**Affiliations:** 1 Department of Internal Medicine, King George's Medical University, Lucknow, IND; 2 Department of Cardiology, King George's Medical University, Lucknow, IND; 3 Department of Respiratory Medicine, King George's Medical University, Lucknow, IND; 4 Department of Otolaryngology - Head and Neck Surgery, King George's Medical University, Lucknow, IND

**Keywords:** polysomnography, obesity, body mass index, hypertension, obstructive sleep apnea

## Abstract

Introduction

About one-half of patients who have essential hypertension have obstructive sleep apnea (OSA), and about one-half of patients who have obstructive sleep apnea have essential hypertension. OSA can cause even resistant hypertension if left untreated. These two entities often co-exist and are seen as a continuum of the same process. Eighty percent to 90% of OSA cases are undiagnosed mostly because of a lack of awareness.

Material and methods

This was a cross-sectional study done over a period of one year in a tertiary care hospital. After taking informed consent, 179 hypertensive patients of >18 years were included in the study. All patients were screened for OSA by the STOP-BANG questionnaire. Patients having scores of ≥3 were subjected to overnight polysomnography to confirm the diagnosis of OSA (AHI ≥5). Patients with a STOP-BANG score ≤2 or score ≥3 with AHI <5, were considered non-OSA.

Results

More than half (53.1%) of the patients enrolled in the study had OSA. Their age ranged from 18 to 78 years and the mean age was 52.07±11.40 years. The mean age of OSA cases was found to be slightly higher than that of non-OSA. The majority of OSA cases were males (73.7%). There was an increase in the prevalence, as well as the severity of OSA, with an increase in BMI. Most of the cases had snoring and a history of tiredness. Triglyceride (TG) and low-density lipoprotein (LDL) levels of the OSA group were found to be significantly higher and high-density lipoprotein (HDL) levels were significantly lower than that of the non-OSA group.

Conclusion

More than half of our hypertensive patients had OSA. These two conditions often co-exist and are known as a dangerous pair. Physicians ought to have increased suspicion for early diagnosis and treatment to improve cardiovascular outcomes, reduce road traffic accidents, and improve quality of life.

## Introduction

Obstructive sleep apnea (OSA) and hypertension (HTN) frequently co-exist and their association is an issue of increasing concern because they are known as a dangerous pair [1,2]. Mild to moderate OSA is present in a considerable proportion of the adult population [3]. Around 50-60% of OSA patients have daytime hypertension, whereas >30% of hypertensive patients were reported to have OSA, and OSA is likely to be an important cause of drug-resistant hypertension [4-8]. Studies have demonstrated a reduction in blood pressure by four to nine weeks of nasal continuous airway pressure in OSA patients [9,10].

In patients with moderate-to-severe OSA, repeated episodes of hypoxia and reoxygenation lead to inflammation and oxidative stress, and accelerate the development of atherosclerosis [11,12]. The exact mechanism behind the susceptibility to hypertension in OSA patients is not clear [12]. Patients with OSA often have impaired social life and depression and are more often involved in accidents. The increased risk of cardiovascular mortality in OSA is independent of other known risk factors such as obesity. OSA may have a synergistic role to play with other traditional and newer risk factors for the development of hypertension [13,14]. Males and females may have different risks due to differential risk factor patterns. This study was conducted with the aim to study the prevalence of OSA in hypertensive patients.

## Materials and methods

Our study was designed as a cross-sectional observational study, conducted in the Department of Medicine at a tertiary care center in Northern India over one year. Hypertensive patients of age ≥ 18 years, willing and able to provide consent, were enrolled in the study. Hypertension was defined according to the European Society of Cardiology (ESC)/European Society of Hypertension (ESH) 2018 criteria - newly diagnosed patients with blood pressure ≥ 140 systolic blood pressure (SBP) and/or ≥90 diastolic blood pressure (DBP) or known cases of hypertension on treatment. Patients with end-stage renal disease (ESRD) and glomerular filtration rate (GFR) < 15 ml/min/m^2^, patients with known chronic respiratory diseases (chronic obstructive pulmonary disease (COPD), asthma), chronic liver disease, coronary artery disease, congestive heart failure, diabetes mellitus, thyroid dysfunction, and acquired immune deficiency syndrome (AIDS), drug abuser, depressive patient, pregnant, alcoholic patients, obvious airway abnormalities, or history of maxillofacial, neck trauma and surgery were excluded from the study.

Methodology

A detailed history regarding his/her age, sex, underlying diseases, smoking, drinking, and exercise was noted and a physical examination was done for all the hypertensive patients that were enrolled in the study. Blood pressure measurement was done by the conventional office blood pressure measurement method using the Heine Gamma G5 Aneroid Blood pressure apparatus (HEINE Optotechnik GmbH & Co., Gilching, Germany). Apart from blood pressure, height, weight, neck circumference, upper arm circumference, and waist circumference were measured for all patients. BMI was calculated and obesity was defined as BMI>25 kg/m^2^. All patients were investigated for complete blood count, serum electrolytes, kidney function test lipid profile, random blood glucose, thyroid profile, and abdominal ultrasonography. All patients were subjected to the eight-item STOP-BANG questionnaire, including Snoring, Tiredness during the daytime, Observed apnea, high blood Pressure, Body mass index, Age, Neck circumference, and Gender (male), for screening for OSA. Each item scored one point. In accordance with previous studies, we used a cut-off value of three, as a score ≥3 has a high sensitivity to detect OSA. Patients having scores≥3 were subjected to full-night polysomnography. The apnea-hypopnea index (AHI) was used to classify OSA into mild (AHI 5-14), moderate (AHI 15-30), and severe (AHI >30).

Statistical analysis

The Statistical Package for Social Sciences (SPSS) version 21.0 statistical analysis software (IBM Corp., Armonk, NY) was used for statistical analysis. The group of continuous variables was compared by the student's t-test for analysis of variance (ANOVA) and discrete data were analyzed using the chi-square test. The values were represented in number (%) and mean±SD. The level of significance p-value of ≤0.5 was considered significant.

Ethical approval

The present study was approved by the institutional ethical committee of King George's Medical University and was not supported by any funding agency.

## Results

A total of 179 hypertensive patients fulfilling the inclusion criteria were enrolled in the study after obtaining their consent. All subjects were administered the STOP-BANG questionnaire to qualify for the polysomnography. Out of 179 cases, 42 (23.5%) cases had a STOP-BANG score ≤2, had very less risk of obstructive sleep apnea, and did not qualify for polysomnography; the rest (137; 76.5%) cases had a STOP-BANG score of ≥3 and were subjected to polysomnography (Table 1).

**Table 1:** STOP-BANG score of the study population (N=179)

STOP-BANG score	No. of cases	Percentage
Score ≤2	42	23.5
Score ≥3	137	76.5

**Figure 1:**
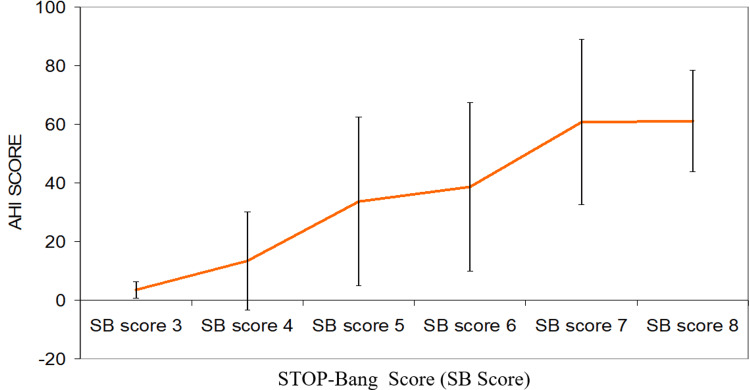
Association of AHI with the STOP-BANG score AHI: apnea-hypopnea index

Out of 137 cases who underwent polysomnography, 42 had an AHI score of <5.0, and the rest (95) had an AHI score of ≥5 (Table 2). Only these 95 cases were diagnosed as OSA. A trend of increase in the AHI score with an increase in the STOP-BANG score was observed. A minimum AHI score was found for cases with the STOP-BANG score of 3 (3.51±2.74), which subsequently increased to 61.08±17.23 for a STOP-BANG score of 8. The association between the AHI score and the STOP-BANG score was found to be significant statistically (Table 3 and Figure 1).

**Table 2:** Polysomnography in hypertensive patients with a STOP-BANG score of ≥3 (n=137) AHI: apnea-hypopnea index

Polysomnography	No. of cases	Percentage
AHI <5.0	42	30.7
AHI ≥5.0	95	69.3

**Table 3:** Association of the STOP-BANG score and AHI score AHI: apnea-hypopnea index

STOP-BANG score	No. of cases	Min. AHI score	Max. AHI score	Mean	±S.D.
3	31	1	16	3.51	±2.74
4	36	1	94.9	13.42	±16.67
5	24	2.8	114.5	33.63	±28.79
6	31	6	145.8	38.64	±28.77
7	10	31.7	111.1	60.69	±28.14
8	5	39.9	84.1	61.08	±17.23
Total	137	1	145.8	25.61	±28.19

After administration of the STOP-BANG questionnaire and thereafter polysomnography of screened cases, 84 hypertensive cases had either a STOP-BANG score of ≤2 or a score of ≥3 but an AHI score <5 was not found to have the risk of OSA. Rest 95 (53.1%) cases having STOP-BANG score ≥3 and AHI score ≥5 were diagnosed as OSA. Thus the prevalence of OSA in hypertensive cases (N=179) was 53.1% (Table 4). The severity of OSA cases is shown in Table 5. Among OSA cases (n=95), 28 (29.5%) had AHI scores of 5 to 14 and were classified as mild, 22 (23.2%) had AHI scores of 15-30 and were classified as moderate, and the rest (45; 47.4%) of the cases having an AHI score of >30 were classified as severe OSA.

**Table 4:** Prevalence of OSA in the hypertensive population (N=179) AHI: apnea-hypopnea index; OSA: obstructive sleep apnea

Group	Description	No. of patients	Percentage
OSA	Hypertensive patients with a STOP-BANG score of ≥3 and AHI ≥5	95	53.1
Non-OSA	Hypertensive patients with a STOP-BANG score of ≤2 or score ≥3 but AHI <5	84	46.9
		179	100.0

**Table 5:** Severity of OSA cases (n=95) AHI: apnea-hypopnea index; OSA: obstructive sleep apnea

Severity	No. of patients	Percentage
Mild (AHI 5-14)	28	29.5
Moderate (AHI 15-30)	22	23.2
Severe (AHI >30)	45	47.4

The age of hypertensive cases in our study ranged from 18 to 78 years and the mean age was 52.07±11.40 years. The mean age of OSA cases (52.34±11.40 years) was found to be slightly higher than that of non-OSA cases (51.73±11.88 years) but this difference was not found to be statistically significant. In lower age groups, i.e. up to 40 years, the proportion of non-OSA cases was higher as compared to OSA cases while among the higher age group, the proportion of OSA cases was higher as compared to non-OSA. This association was also not found to be significant statistically. Out of 179 cases enrolled in the study, 110 (61.5%) were males and the rest were females. The majority of the OSA cases were males (73.7%) while the majority of non-OSA cases were females (52.4%). This difference was found to be significant statistically. No significant association of severity of OSA was observed either with age or gender. OSA cases, as compared to non-OSA hypertensive cases, had significantly higher height, weight, and BMI. However, the association between the severity of OSA and anthropometric parameters was not found to be significant statistically (Table 6).

**Table 6:** Association of anthropometric parameters with OSA OSA: obstructive sleep apnea

Parameters	OSA (n=95)		Non-OSA (n=84)		Significance of differences	
	Mean	±SD	Mean	±SD	‘t’	‘p’
Height	161.45	±8.57	157.93	±8.76	2.718	0.007
Weight	84.45	±17.17	64.26	±12.00	9.004	<0.001
BMI	32.61	±6.57	25.82	±4.12	8.148	<0.001

Out of 179 hypertensive cases, 138 were overweight (BMI >25 kg/m^2^). Out of 138 overweight patients, 89 belonged to the OSA group and 49 to the non-OSA group. A subsequent increase in the proportion of overweight patients was observed with an increase in the severity of OSA, i.e. mild (85.7%), moderate (90.9%), and severe (100.0%). This association was found to be significant statistically (p=0.042). The proportion of non-OSA cases was higher as compared to OSA cases with lower BMI, i.e. <25 kg/m^2^ (42.9% vs. 6.3%) and 25-30 kg/m^2^ (42.9% vs. 32.6%) while the proportion of OSA cases was higher as compared to non-OSA having a higher BMI, i.e. 30.1-35 kg/m^2^ (31.6% vs. 11.9%) and BMI >35 kg/m^2^ (29.5% vs. 2.4%). This difference was found to be statistically significant (p <0.001).

The proportion of severe OSA cases was higher as compared to mild and moderate OSA cases having BMI >35 kg/m^2^ (40.0% vs. 21.4% & 18.2%) while the proportion of mild and moderate OSA cases was higher as compared to severe OSA having BMI <25 kg/m^2^ (14.3% & 9.1% vs. 0.0%), BMI 25-30 kg/m^2^ (35.7% & 36.4% vs. 28.9%), the rest of the cases had BMI 30.1-35.0 kg/m^2^. The association between BMI and the severity of OSA was not found to be significant statistically (p=0.134).

Out of 179 hypertensive cases, only three (1.7%) were not taking any treatment for hypertension, as they were newly detected and all cases were having OSA. Out of 176 taking medications, the majority (93.75%) of them had controlled hypertension. The proportion of cases with uncontrolled hypertension was higher among OSA as compared to non-OSA (11.5% vs. 1.2%). This difference was found to be statistically significant (p=0.017). Uncontrolled hypertension cases were having a significantly higher proportion of cases of severe OSA as compared to mild and moderate OSA (20.0% vs. 3.6% & 0.0%) (p=0.016). Patients having OSA were taking more medications for hypertension than non-OSA cases but the difference was not found to be statistically significant. The association of the number of medications with the severity of OSA was also not found statistically significant. Though the duration of hypertension was higher among non-OSA cases (5.81±5.02 years) as compared to OSA cases (5.30±4.54 years), this difference was not found to be statistically significant (p=0.477).

All the OSA cases had a history of snoring as compared to only 36.9% of non-OSA; this difference was found to be significant statistically (p<0.001). Only 14.7% of OSA cases and 82.1% of non-OSA cases felt energetic, the rest complained of tiredness. The difference in the proportion of cases with complaints of tiredness was significantly higher in the OSA group as compared to the non-OSA group (85.3% vs. 17.9%). Complaints of tiredness were observed in a higher proportion of moderate and severe OSA patients as compared to mild OSA (95.5% & 95.6% vs. 60.7%); this difference was found to be significant statistically (p<0.001).

Significant differences among OSA and non-OSA cases were observed for triglyceride, high-density lipoprotein (HDL), and low-density lipoprotein (LDL) levels. The HDL levels of OSA cases were found to be significantly lower than that of non-OSA cases. Triglyceride and LDL levels in OSA cases were found to be significantly higher than that in non-OSA cases. No significant association between the severity of OSA and the above parameters was found.

## Discussion

Obstructive sleep apnea (OSA) is a highly prevalent sleep disorder affecting the majority of the adult population. The major risk factors for OSA are obesity, male sex, advancing age, and elevated blood pressure. OSA is a potential cause of resistant hypertension. A major portion of hypertensive patients have concomitant OSA, but they usually remain undiagnosed due to a lack of awareness and inadequate screening. Most of the studies related to the prevalence of OSA in hypertension are from outside India [14].

In the present study, we screened 179 hypertensive cases using the STOP-BANG questionnaire for OSA. Out of these, 137 (76.5%) cases having a STOP-BANG score of ≥3 were subjected to polysomnography. Out of those who underwent polysomnography, 42 (30.7%) had an AHI score of <5.0, and the rest 95 (69.3%) had an AHI of ≥5. Only these 95 cases were diagnosed as OSA. Thus, the prevalence of OSA in our hypertensive patient was 53.1%. A trend of increase in the AHI score with an increase in the STOP-BANG score was observed. The association between the AHI score and the STOP-BANG score was found to be significant statistically. A similar study was conducted by Ricardo Luiz de Menezes Duarte et al. (2017). In their study, they found that an increase in the score was paralleled by an increase in specificity for all AHI cut-off points. According to their study, the STOP-BANG questionnaire is the best screening test for OSA [15]. A cross-sectional analysis by Elizabeth S. Muxfeldt et al. was of 422 resistant hypertension (RHT) (31.3% men; mean age = 62.4 ± 9.9 years) submitted to full-night polysomnography. RHT patients had a very high prevalence of OSA (82%) and moderate/severe OSA (55%) [16].

The mean age of the patients enrolled in our study was 52+11.4 years. The mean age of the OSA cases (52.34+11.40) was slightly higher than non-OSA cases (51.73+11.88). In the study by Cristopher John Worsnop et al., the mean age of the patients was 58+2.1 and 60.9+2 among untreated hypertensives and treated hypertensives, respectively, which correlates with our study [5]. Our study showed that higher age is a significant risk factor for OSA.

Out of 179 cases enrolled in the study, 110 (61.5%) were males and the rest were females. The majority of the OSA cases were males (73.7%) while the majority of non-OSA cases were females (52.4%). This difference was found to be significant statistically. This is supported by Vahid Mohsenin et al., who studied 736 OSA cases, in which males were more than females. This study was also supported by the study of Hedner et al. on 141 patients with hypertension in primary health care, which showed a higher prevalence of OSA in males than females, concluding that there was an independent association between hypertension and OSA in males but not in females [17,18].

Out of 179 hypertensive cases, 138 (93.7% of OSA and 58.3% of non-OSA cases) were overweight (BMI >25 kg/m^2^). The mean weight of the OSA group (84.45±17.17 kg) had a significantly higher weight as compared to the non-OSA group (64.26+12.00 kg). An increase in the proportion of overweight patients was also observed with an increase in the severity of OSA, i.e. mild (85.7%), moderate (90.9%), and severe (100.0%). In the study, OSA is associated with higher BMI, and the severity of OSA also increases with an increase in BMI. Thus, it can be esteemed that the prevalence, as well as the severity of OSA, increases with increasing BMI. This was supported by Gary D. Foster. et al, in whose study over 86% of participants had OSA with an AHI of ≥5 events/h. The mean AHI was 20.5 ± 16.8 events/h. Severe OSA was more in individuals with a higher BMI (odds ratio 1.1; 95% CI 1.0-1.2; P = 0.03) [19].

Out of 179 hypertensive cases, only three (1.7%) were not taking any treatment for hypertension, as they are newly diagnosed and all three cases had OSA. Out of 176 taking medications, the majority (93.75%) of them had controlled hypertension. The proportion of cases with uncontrolled hypertension was higher among OSA as compared to non-OSA (11.5% vs. 1.2%). This difference was found to be significant statistically (p=0.017). Most of these uncontrolled hypertension cases had a significantly higher proportion of cases of severe OSA as compared to mild and moderate OSA (p=0.016). Patients who had OSA were taking more medications for hypertension than non-OSA cases but the difference was not found statistically significant. The association of the number of medications with the severity of OSA was also not found to be significant statistically.

In a cross-sectional community-based study by Khin Mae Hla et al., the measurement of blood pressure during wakefulness and sleep among participants with and without sleep-disordered breathing. A total of 147 men and women, aged 30 to 60 years, selected from Wisconsin State employees were enrolled in the Wisconsin Sleep Cohort Study. Mean blood pressures were significantly higher among participants with sleep apnea compared with those without. The variability of the blood pressure during sleep was significantly greater in participants with sleep apnea or a history of snoring as compared with those without (P < 0.05) [14]. Also supported by Christopher John Worsnop et al., 38% of the 34 untreated, 38% of the 34 treated hypertensives, and 4% of the 25 normotensives had AHI ≥5. Logistic regression analysis showed that treated hypertension (p= 0.05) and untreated hypertension (p= 0.06) were associated with the presence of sleep apnoea; they concluded that there is a relationship between sleep apnoea and hypertension [5].

Out of 10 cases of uncontrolled hypertension/resistant hypertension (RHT), nine patients had OSA, which is significantly associated with severe OSA (p<0.016) as compared to mild or moderate OSA, which is supported by Pimon Ruttanaumpawan et al. who compared the prevalence of OSA in 42 patients with RHT with 22 patients with controlled hypertension, the RHT group had a significantly higher prevalence of OSA (81 versus 55%, P=0.03). Multivariate analysis revealed significantly increased odds of having RHT (adjusted odds ratio, 3.994; 95% confidence interval, 1.191-13.388) [20].

In our study, all the OSA groups had a history of snoring as compared to only 36.9% of the non-OSA group. This difference was found to be significant statistically (p<0.001). Our study is supported by Thikriat S. Al-Jewair et al., who studied a total of 200 consecutive female and male dental patients. Habitual snoring was present in 18.2% of the females and 81.8% of the males (p<0.05). Breathing pauses during sleep more than once a week occurred in 9% (n=17) of the sample. Of the males, 78.3% were at high risk of OSA as compared with 21.7% of the females [21].

## Conclusions

More than half of the patients enrolled in the study had OSA. Male patients who had increased weight, BMI, and uncontrolled hypertension have a much more chance of developing severe OSA. As the prevalence of OSA is significantly higher among hypertensive patients, these patients should be screened easily by a simple questionnaire and subsequently may be subjected to polysomnography for early diagnosis and treatment to reduce morbidity and mortality and improve the quality of life.
